# Trends in In-Hospital Mortality among Patients with Stroke in China

**DOI:** 10.1371/journal.pone.0092763

**Published:** 2014-03-20

**Authors:** Qian He, Cheng Wu, Hong Luo, Zhi-Yong Wang, Xiu-Qiang Ma, Yan-Fang Zhao, Jian Lu, Chun Xiang, Ying-Yi Qin, Shun-Quan Wu, Fei-Fei Yu, Jia He

**Affiliations:** 1 Department of Health Statistics, Second Military Medical University, Shanghai, China; 2 Center of Educational Technology, Second Military Medical University, Shanghai, China; 3 Department of Information, Changhai Hospital, Second Military Medical University, Shanghai, China; Kaohsiung Chang Gung Memorial Hospital, Taiwan

## Abstract

**Background:**

The incidence and burden of stroke in China is increasing rapidly. However, little is known about trends in mortality during stroke hospitalization. The objectives of this study were to assess trends of in-hospital mortality among patients with stroke and explore influence factors of in-hospital death after stroke in China.

**Methods:**

109 grade III class A hospitals were sampled by multistage stratified cluster sampling. All patients admitted to hospitals between 2007 and 2010 with a discharge diagnosis of stroke were included. Trends in in-hospital mortality among patients with stroke were assessed. Influence factors of in-hospital death after stroke were explored using multivariable logistic regression.

**Results:**

Overall stroke hospitalizations increased from 79,894 in 2007 to 85,475 in 2010, and in-hospital mortality of stroke decreased from 3.16% to 2.30% (*P*<0.0001). The percentage of severe patients increased while odds of mortality (2010 versus 2007) decreased regardless of stroke type: subarachnoid hemorrhage (*OR* 0.792, 95% *CI* = 0.636 to 0.987), intracerebral hemorrhage (*OR* 0.647, 95% *CI* = 0.591 to 0.708), and ischemic stroke (*OR* 0.588, 95% *CI* = 0.532 to 0.649). In multivariable analyses, older age, male, basic health insurance, multiple comorbidities and severity of disease were linked to higher odds of in-hospital mortality.

**Conclusions:**

The mortality of stroke hospitalizations decreased likely reflecting advancements in stroke care and prevention. Decreasing of mortality with increasing of severe stroke patients indicated that we should pay more attention to rehabilitation and life quality of stroke patients. Specific individual and hospital-level characteristics may be targets for facilitating further declines.

## Introduction

Stroke is one of the leading causes of death and disability throughout the world [1], responsible for 4.4 million (9%) of the total 50.5 million deaths each year [2]. The incidence and burden of stroke in China is increasing rapidly over time just like in other developing countries. About 2 million people of all ages suffer a new stroke, and 15 million stroke-related deaths occur in each year [3]. It is now becoming the first leading cause of death in China [1]. Mortality of stroke at discharge significantly increased with age, with 1.15%, 1.46%, 3.31%, and 7.63% in-hospital mortality according to age group (≤45,46–65,66–79, ≥80 years old) respectively, and the very old patients had the worst outcomes even after adjusted by prognostic factors[4]. Now, demographic ageing is occurring at an unprecedented rate worldwide; the proportion of Chinese aged 65 and over will increase from 4% in 2000 to 14% by 2025, amounting to 200 million old people [5]. Aging may also result in an increased risk for stroke [6], which produces more and more burden on society and families [1]. It becomes very urgent to understand the trend of stroke prognosis. However, recent information about in-hospital mortality trends after stroke hospitalization is lack in China, though historical and risk factors of mortality after stroke have been identified in various studies including clinical trials, community-based studies, and voluntary registries[4], [7]. With population aging, the health reform was deepened and stroke prevention, treatment and rehabilitation care were promoted continually. Knowledge of in-hospital deaths after stroke may be helpful for knowing the “real-world” and interface of challenges in optimizing overall premorbid health status, stroke prevention, acute stroke treatment, and acute general medical care at the individual, hospital, and health system levels [7].

Analyzing of influence factors of trend of stroke outcomes may lead to strategies for enhancing quality improvement at several levels of policymaking [7].The objectives of this study were to assess trends of the proportion of stroke hospitalizations that resulted in death in China in recent years and find some potential influence factors of in-hospital mortality after stroke hospitalization.

## Methods

### Setting, sampling and study design

Using a multistage stratified cluster sampling method,109 grade III (third tier) class A hospitals were enrolled representing 765 grade III class A hospitals in China. They were from the northern (10 provinces), eastern (4 provinces), southern (7 provinces), western (10 provinces) China respectively. The hospitals in China were classed to 9 types: grade III class A, grade III class B, grade III class C, grade II class A, grade II class B, grade II class C, grade I class A, grade I class B, and grade I class C. The highest rank is the Grade III class A hospital, which is a large-scale general hospital integrating medical service, education and research including more than 500 beds. From 2007 to 2010, the discharges from sampled hospitals for the calendar year were selected into this study.

### Data collection and analysis

To identify stroke hospitalizations according to the primary diagnosis, we used all discharges for which International Classification of Diseases, 9th Revision (ICD-9) codes 430 to 438 in primary diagnosis for conservative estimation [7], [8]. When analyzing based on hospital diagnosis statistics, generally they count cases, not patients [7]. A person treated several times at hospital will be counted multiply. 350 036 discharges that had stroke from January 2007 to December 2010 were recruited.

Data were captured including demographics, discharge-level information on diagnoses, primary payer (self-payment, basic health insurance, other), comorbid condition, severity of disease (mild, moderate and severe), results of treatment, length of stay in hospital (LOS) and hospital region (northern, eastern, southern and western China). We considered all discharges to be independent.

The study was approved by the Ethics Committee of the Second Military Medical University, Shanghai, China. The ethics committee waived the need of informed consent for the study because of its retrospective nature and data were analyzed anonymously.

### Statistical Analysis

Comparing baseline characteristics between 2007 and 2010, numerical variables which followed normal distribution were analyzed by ANOVA, non-normal distribution were analyzed by Kruskal–Wallis test, such as age and LOS. Unordered categorical variables were analyzed by chi-square test or Fisher's exact test, including gender, primary payer, hospital region; ordinal categorical variables were analyzed by Kruskal–Wallis test including Charlson comorbidity index (CCI) and severity of disease.

Trends of in-hospital mortality after stroke were analyzed across time stratified by stroke type and hospital region. Stroke type was categorized as follows [6], [7], [9]: (1) subarachnoid hemorrhage (SAH; ICD-9 430); (2) intracerebral hemorrhage (ICH; ICD-9 431); (3) ischemic stroke (IS; ICD-9 433, 434, and 436). To assess linear trend of in-hospital mortality from year 2007 to 2010, the Cochran-Armitage trend test was used. To assess adjusted in-hospital mortality trends and identify independent predictors of in-hospital mortality, the multivariable logistic regression model was used. The following demographic and clinical characteristics were adjusted: age, gender, primary payer, comorbid condition, severity of disease, and LOS. In addition, the hospital region was also adjusted for. Year was involved as a categorical variable which was transformed to dummy variables in the model.

The severity of disease was classified into three groups by National Institutes of Health Stroke Scale (NIHSS), and they were mild (≤6), moderate (≥7 and ≤14) and severe (≥15). The number and severity of comorbid conditions were assessed using the Charlson comorbidity index (CCI). The modified version of the CCI was used^7^. The CCI is a weighted score composed of 17 comorbid conditions including congestive heart failure (weight 1), myocardial infarction (weight 1), chronic pulmonary disease (weight 1), cerebrovascular disease (weight 1), hemiplegia or paraplegia (weight 2), dementia (weight 1), diabetes without complications (weight 1), diabetes with complication (weight 2), malignancy (weight 2), metastatic solid tumor (weight 6), mild liver disease (weight 1), moderate or severe liver disease (weight 3), peptic ulcer disease (weight 1), peripheral vascular disease (weight 1), rheumatologic disease (weight 1), renal disease (weight 2), and AIDS (weight 6). In the multivariable analysis, CCI was grouped into 4 categories [7], including a CCI of 1, 2, 3, or ≥4.

All tests were 2-tailed, and *P*≤0.05 was considered significant. Statistical analysis was performed using a commercially available software package (SAS statistical software version 9.3; SAS Institute Inc., Cary, NC, USA).

## Results

**Figure 1:**
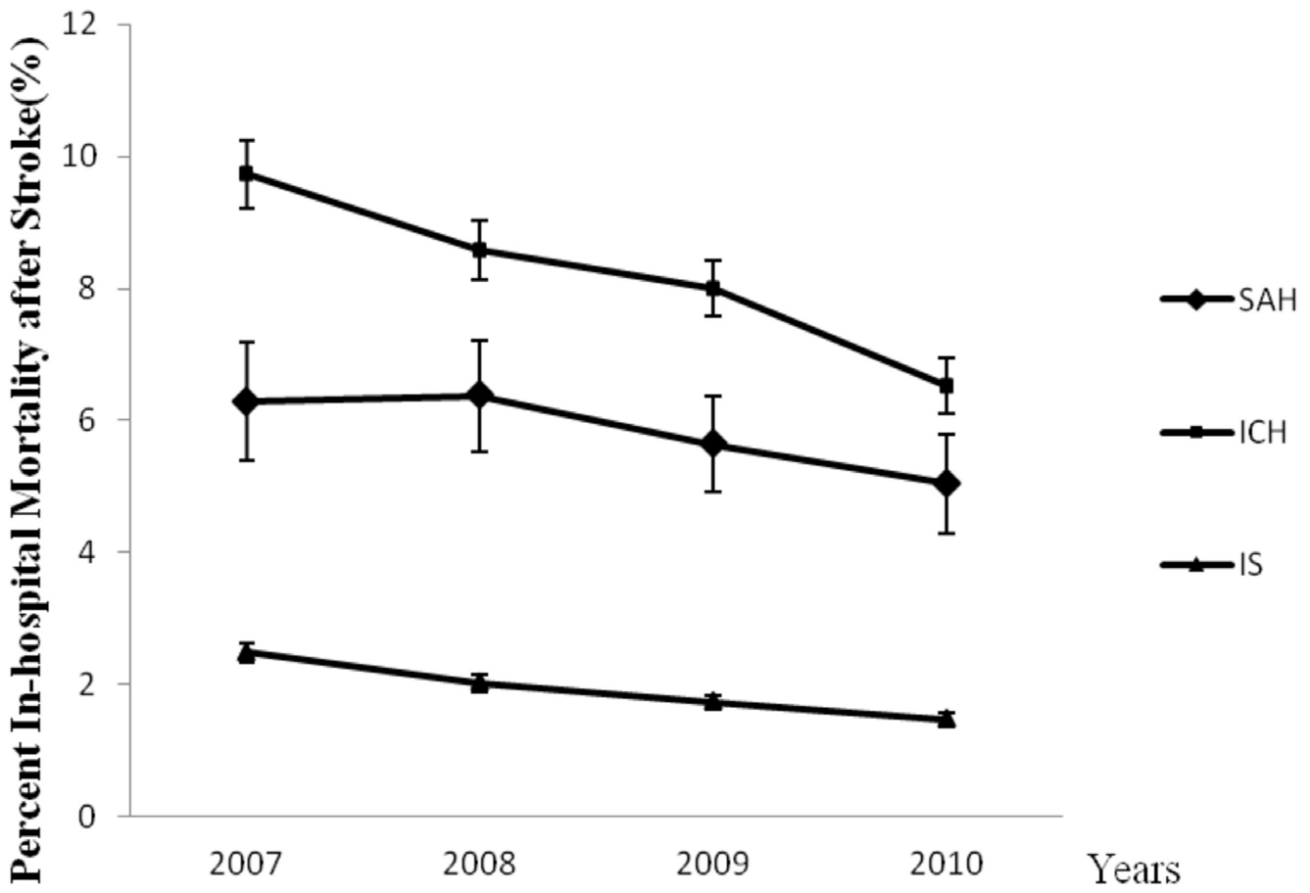
In-Hospital Mortality after Stroke by Type between 2007 and 2010 in China.

Overall, stroke hospitalizations increased from 79 894 in 2007 to 85 475 in 2010 in the 109 grade III class A hospitals, whereas overall percentage of stroke hospitalizations that resulted in death decreased from 3.16% to 2.30% (*P*<0.0001). Over these years, the mortality of stroke inpatients declined steadily, a trend generally seen across stroke types (Figure 1). Table 1 shows the summary for demographic, clinical, and regional factors for each stroke types. From 2007 to 2010, there was a modest but significant increase in age for SAH (*P* = 0.0054) and IS (*P*<0.0001), but not for ICH (*P* = 0.2572). And compared with 2007, there were more males in 2010 for IS (*P* = 0.0211) but not for SAH or ICH (*P* = 0.8438 or 0.2562). During these years, primary payer changed significantly for all stroke types (*P*<0.0001), and there were more patients who paid by basic health insurance while fewer self-payment. The CCI became less by year (*P*<0.0001), and the proportion of severe status was increased (*P*<0.0001). The number of inpatient increased in north for SAH (*P*<0.0001), west for ICH (*P*<0.0001), south and west for IS (*P*<0.0001). The LOS of IS decreased (*P*<0.0001) while ICH increased (*P* = 0.0010), and the change of SAH was not significant.

**Table 1:** Descriptive Socio-demographic and Clinical Characteristics of Hospitalized Patients in China with a Diagnosis of Stroke between 2007 and 2010 by Stroke Type.

	SAH				ICH				IS			
Variables	2007		2010		2007		2010		2007		2010	
	(*N* = 2787)		(*N* = 3214)		(*N* = 12841)		(*N* = 13451)		(*N* = 38418)		(*N* = 45641)	
Demographic factors												
Age, years	53	43–62	54	45–63	58	49–69	58	49–69	68	57–75	68	58–76
Median, *Q*_1_–*Q*_3_	53	43–62	54	45–63	58	49–69	58	49–69	68	57–75	68	58–76
<45	803	28.82	761	23.68	2142	16.68	2060	15.31	2163	5.63	2161	4.73
45–64	1397	50.14	1757	54.67	6143	47.84	6709	49.88	13629	35.48	17132	37.57
65–74	381	13.68	444	13.81	2760	21.49	2624	19.51	12358	32.17	13141	28.79
75–79	120	4.31	147	4.57	999	7.78	1151	8.56	5566	14.49	6905	15.13
80	85	3.05	105	3.27	797	6.21	907	6.74	4702	12.24	6302	13.81
Sex												
Male	1317	47.27	1506	46.86	8037	62.59	8539	63.48	23129	60.20	27557	60.38
Female	1469	52.73	1708	53.14	4804	37.41	4912	36.52	15288	39.80	18084	39.62
Primary payer												
self-payment	1890	67.84	1659	51.62	8586	66.86	6802	50.57	19581	50.97	16601	36.37
basic health insurance	704	25.27	1362	42.38	3718	28.95	6193	46.04	16674	43.40	27062	59.29
other	192	6.89	193	6.00	537	4.19	456	3.40	2163	5.63	1978	4.34
CCI												
1	2431	87.29	2865	89.14	11064	86.16	12220	90.85	26790	69.73	35184	77.89
2	312	11.20	320	9.96	1311	10.21	795	5.91	9761	25.41	9093	19.92
3	35	1.26	26	0.81	383	2.98	364	2.71	1314	3.42	1019	2.23
≥4	7	0.25	3	0.09	83	0.65	72	0.53	553	1.44	345	0.76
Severity of disease												
severe	77	2.76	198	6.16	330	2.57	559	4.16	157	0.42	348	0.76
moderate	1173	42.10	1451	45.15	5689	44.30	6021	44.76	10011	26.06	11547	25.30
mild	1536	55.14	1565	48.69	6822	53.13	6871	51.08	28250	73.52	33746	73.94
Hospital region												
North (10 provinces)	950	34.10	1097	34.13	5446	42.41	5455	40.55	19486	50.72	22683	49.70
East (4 provinces)	579	20.78	610	18.98	1776	13.83	2050	15.24	5191	13.51	5271	11.55
South (7 provinces)	511	18.34	558	17.36	2166	16.87	2232	16.59	4976	12.95	6556	14.36
West (10 provinces)	746	26.78	949	25.53	3453	26.89	3714	27.62	8765	22.82	11131	24.39
Length of stay												
Days (Median, *Q*_1_–*Q*_3_)	12	5–20	12	5–19	14	6–23	15	7–24	13	9–18	12	8–16

From 2007 to 2010, SAH, ICH and IS hospitalizations increased by 15.32%, 4.75% and 18.80% respectively. In-hospital mortality rates across time for each stroke types are shown in Table 2 and Figure 1. There was a decrease in the in-hospital mortality of SAH (from 6.28% to 5.04%; *P* = 0.0167), ICH (from 9.73% to 6.52%; *P*<0.0001) and IS (from 2.48% to 1.47%; *P*<0.0001). There were similar trend in northern, eastern, southern and western China for ICH and IS (Figure S1). But for SAH mortality decreased significantly in southern and western China, while the mortality in north was the highest. Comparisons of the decrease in in-hospital mortality across time among the different types of stroke are listed in Table 3 (ORs for the effect of year).

**Table 2:** Distribution of In-Hospital Mortality Rates across Time by Stroke Type and Region.

Diagnosis	Year	Overall (%)	North (%)	East (%)	South (%)	West (%)
SAH						
	2007	6.28	6.63	4.84	6.26	6.97
	2008	6.37	7.56	4.68	7. 87	5.00
	2009	5.64	7.03	4.27	5.90	4.75
	2010	5.04	7.57	3.44	3.23	4.21
	Z statistic	−2.393	0.5808	−1.2429	−2.4706	−2.445
	*P* value	0.0167	0.5614	0.2139	0.0135	0.0145
ICH						
	2007	9.73	10.98	9.29	9.51	8.14
	2008	8.58	9.12	8.01	8.75	7.91
	2009	8.00	8.99	6.23	8.89	6.93
	2010	6.52	7.52	5.37	6.45	5.74
	Z statistic	−9.5578	−5.9840	−5.1693	−3.3775	−4.3044
	P value	<0.0001	<0.0001	<0.0001	0.0007	<0.0001
IS						
	2007	2.48	2.49	3.10	2.11	2.32
	2008	2.02	2.02	2.15	1.97	1.98
	2009	1.73	1.97	1.78	1.27	1.49
	2010	1.47	1.66	1.84	1.19	1.10
	Z statistic	−11.1168	−5.8082	−4.6052	−4.7562	−7.2555
	*P* value	<0.0001	<0.0001	<0.0001	<0.0001	<0.0001
Overall						
	2007	3.16	3.24	3.62	3.35	2.69
	2008	2.94	2.91	3.09	3.36	2.67
	2009	2.79	3.00	2.52	2.85	2.54
	2010	2.30	2.50	2.38	2.12	2.03
	Z statistic	−10.6863	−5.5016	−6.0191	−6.3088	−4.4895
	*P* value	<0.0001	<0.0001	<0.0001	<0.0001	<0.0001

**Table 3:** ORs for the Effect of Year on In-Hospital Mortality for Each Stroke Type.

Stroke Type	OR* (95% CI)	*P* value
SAH	0.792 (0.636, 0.987)	0.0377
ICH	0.647 (0.591, 0.708)	<0.0001
IS	0.588 (0.532, 0.649)	<0.0001

* odds ratio of 2010 versus 2007, taking year 2007 as the reference group.

Table 4 shows the adjusted logistic regression analyses for each stroke type. For hospitalizations with SAH or ICH, older age, basic health insurance payment (versus self-pay), higher CCI, severe status, hospitals located in the northern and southern region (versus western) were significantly associated with higher odds ratios of dying in the hospital, whereas later year, female and LOS were associated with a significant decrease in the risks of in-hospital death. For hospitalizations with IS, older age, basic health insurance (versus self-pay), higher CCI, severe status, hospitals located in the northern region (versus western) were significantly associated with increasing in the risks of dying in the hospital, while later years, and LOS were associated with a significant decrease in the risks of in-hospital mortality. The OR estimates for the effect of year were unaltered after including covariates in the models.

**Table 4:** Logistic Regression Analyses of Influence Factors of In-Hospital Mortality after Stroke from 2007 to 2010 in China.

	SAH		ICH		IS	
Variables	OR (95%CI)	*P* value	OR (95%CI)	*P* value	OR (95%CI)	*P* value
Years						
2008 vs. 2007	0.93 (0.75,1.16)	0.5350	0.83 (0.76,0.90)	<0.0001	0.78 (0.71,0.86)	<0.0001
2009 vs. 2007	0.83 (0.67,1.02)	0.0795	0.74 (0.68,0.81)	<0.0001	0.66 (0.60,0.72)	<0.0001
2010 vs. 2007	0.67 (0.53,0.84)	0.0006	0.59 (0.54,0.65)	<0.0001	0.55 (09.49,0.60)	<0.0001
Demographic factors						
Female vs. Male	0.81 (0.69,0.94)	0.0066	0.75(0.70,0.80)	<0.0001	1.01(0.94,1.08)	0.8194
Age (per year)	1.04(1.04,1.05)	<0.0001	1.02(1.01,1.02)	<0.0001	1.06(1.05,1.06)	<0.0001
Primary payer						
basic health insurance vs. self-pay	1.69(1.43,1.99)	<0.0001	1.82(1.70,1.94)	<0.0001	1.43(1.32,1.54)	<0.0001
other vs. self-pay	0.88(0.61,1.27)	0.4982	1.35(1.16,1.58)	0.0001	1.31(1.11,1.54)	0.0012
CCI						
2 vs. 1	2.68(0.97,7.34)	0.0561	2.88(2.19,3.80)	<0.0001	3.00(2.43,3.70)	<0.0001
3 vs. 1	2.60(1.56,4.34)	0.0003	2.30(2.00,2.65)	<0.0001	2.54(2.22,2.90)	<0.0001
≥4 vs. 1	1.49(1.19,1.88)	0.0007	1.25(1.13,1.39)	<0.0001	0.94(0.86,1.03)	0.1753
Severity of disease						
severe vs. mild	1.69(1.20,2.37)	0.0025	1.39(1.19,1.63)	<0.0001	5.06(3.95,6.50)	<0.0001
moderate vs. mild	1.22(1.04,1.43)	0.0171	1.25(1.17,1.33)	<0.0001	1.85(1.72,1.98)	<0.0001
Hospital region						
North vs. west	1.33(1.09,1.62)	0.0055	1.40(1.30,1.52)	<0.0001	1.17(1.07,1.28)	0.0005
East vs. west	0.81(0.62,1.05)	0.1188	1.02(0.92,1.14)	0.6739	1.01(0.89,1.13)	0.9310
South vs. west	1.32(1.03,1.69)	0.0269	1.41(1.28,1.56)	<0.0001	1.01(0.89,1.15)	0.8639
Length of stay	0.94(0.93,0.95)	<0.0001	0.94(0.94,0.94)	<0.0001	0.98(0.98,0.98)	<0.0001

## Discussion

This analysis of proportions of in-hospital mortality after stroke from 2007 to 2010 in China showed that deaths during stroke hospitalization have lessened significantly over time and since 2008 the decline has been steady and continuous, probably mainly reflecting improvements in hospital care after occurrence of a stroke. The trend of decrease in the percentage of stroke hospitalizations resulting in death is consistent with the observational study from the Sino-MONICA-Beijing from 1984 to 2004 [10] and the crude mortality of cerebrovascular disease from the fourth health service survey in China [11]. The proportion of IS subtype were the largest, followed by ICH and SAH in the hospitals, and these data are generally in accordance with the incidence of stroke in the survey based on community population and the China National Stroke Registry (CNSR) from 2003-2008 in China [12]–[15]. And the proportion of male was higher than female in IS and ICH,which was similar with other studies [7], [12], [15], while the proportion of female was higher than male in SAH, which was similar with Beijing and Shanghai in China from 1991–2000 [16]. The median age was 68 years old among IS patients while less than 60 years old among ICH and SAH patients, and age difference among subtypes also appeared in some community population [13]. There was a good representativeness for stroke patients because of the similar distribution of subtype, gender and age with the observational studies based on community population and CNSR. This study showed that the mortality decreased from 3.16% in 2007 to 2.30% in 2010 (SAH: form 6.28% to 5.04%, ICH: 9.73% to 6.52%, IS: from 2.48 to 1.47%) while the number of patients increased from 79 894 to 85 475. The decrease trend was also found in the United States (SAH: from 26.90% in 1997–1998 to 23.80% in 2005–2006, ICH: from 30.47% to 28.23%, IS: from 9.76% to 8.78%) [7] and in Germany (from 11.9% in 2005 to 9.5% in 2010) [17]. The in-hospital mortality in this study was nearly equal to the data from the China National Registry [18], but lower than Germany and the United States. There may be some reasons. Firstly, the age of patients in this study was younger (this study: 65 vs. Germany: 73 years old). Secondly, the proportion of ICC≥4 which reflects severe level of stroke was very low compared to America (this study in 2007 vs America in 2005–2006: SAH 0.25 vs 10.2, ICH 0.65 vs 24.3, IS 1.44 vs 29.4). Thirdly, patients were earlier transferred to smaller regional hospitals or home. Fourthly, repetitive admission would decrease the mortality. The decreasing of mortality in the United States was likely driven by revascularization strategies among ischemic strokes and better acute stroke care in addition to prevention of stroke [7]. Some health reform strategy and mass approaches on decreasing mortality were also adopted in China including stroke prevention, various unconventional local therapeutic traditions, and several national guidelines on stroke prevention and treatment [11], [19], [20].

On the other hand, the construction of health insurance system was accelerated under the health reform policy and the proportion of basic health insurance became higher through these years. It helped with decreasing catastrophic healthcare payments and more stroke patients gained positive treatment [21]. Severity and CCI were the risk factors for stroke [7]. There were more patients in the northern and western regions. For the different stroke types, more SAH patients appeared in the north, more ICH in the west, and more IS in the south and west. It is partly due to different climates and lifestyles [20]. The mortality of all subtypes of stroke was higher in the north, though it decreased significantly except for SAH.

The change of LOS among SAH patients was no significant, and the LOS among ICH became longer while IS shorter. Mortality of ICH was the highest among all subtypes and needed more time to recover. The health insurance strategy limited the LOS in hospitals due to cost containment, and the IS patients would be transferred to other community hospitals for further recovery. The mortality of ICH was highest, followed by SAH and IS, and decreasing trend was significant in SAH and IS over the time. ORs for the effect of time 2010 versus 2007 on in-hospital mortality for each stroke subtype also showed that the percentage of stroke hospitalizations resulting in death decreased in China, similar with the United States [7].

The multivariable analyses investigated the independent influence factors of dying in the hospital after a stroke, and showed that several socio-demographics and region factors could be further explored to possibly boost survival among hospitalized patients. Many of these factors were similar across stroke types, which included older age, male, northern and western regions and which had previously been associated with larger risk of short-term mortality after stroke, and underscored the need to implement strategies to bridge these socio-demographic gaps in stroke care [4], [14], [20]. In this study, patients whose primary payer was the basic health insurance had more odds of mortality in hospital. Patients would choose treatment in hospital much more easily and not care the high expenditure if they had the basic health insurance. Generally patients in worse condition were more likely to die during stroke hospitalization. However, it was surprising to observe that the number of hospitalized stroke patients in server condition has risen over these years. It may be because that condition of patients are progressively getting worse, but a more likely explanation in the face of declining in-hospital mortality rates after stroke could be that better diagnostic techniques and better awareness have led to increased diagnoses of several general medical conditions over the years. Considering on decreasing of mortality and increasing of number of worse stroke patients, the rehabilitation and life quality of stroke patients should be paid more attentions in China. Since 2009 national screening, prevention and control of stroke project was promoted. The CCI became lower, which is inconsistent with the United State [7].

This study has limitations. Because of the hospitals were grade III class A, this analysis would not reflected other grade hospitals completely though the gender, age, subtype distribution were the same with the survey and community population. So earlier transfer of patients to smaller regional hospitals would bias the numbers of intra-hospital mortality.

Other limitations include inability to distinguish first from recurrent strokes, and lack of documented information on the rate of non-hospitalized stroke in the country because many stroke-related deaths occurred out of the hospital. The study was strengthened by its nationwide scope, incident and not prevalent stroke rate data, clinician-diagnosed and not self-reported strokes. The time-interval may be a little short in this study, further research should be taken.

## Supporting Information

Figure S1 **In-Hospital Mortality after Stroke by Type between 2007 and 2010 in the Different Regions.** Data presented trends of in-hospital mortality and its 95% CI in different years of three stroke types including SAH, ICH and IS.
(TIF)
