# Supplementary figures and images for: Trends in In-Hospital Mortality among Patients with Stroke in China
Source: PLoS One. 2014 Mar 20;9(3):e92763. doi: 10.1371/journal.pone.0092763 (PMC3961404; doi:10.1371/journal.pone.0092763)

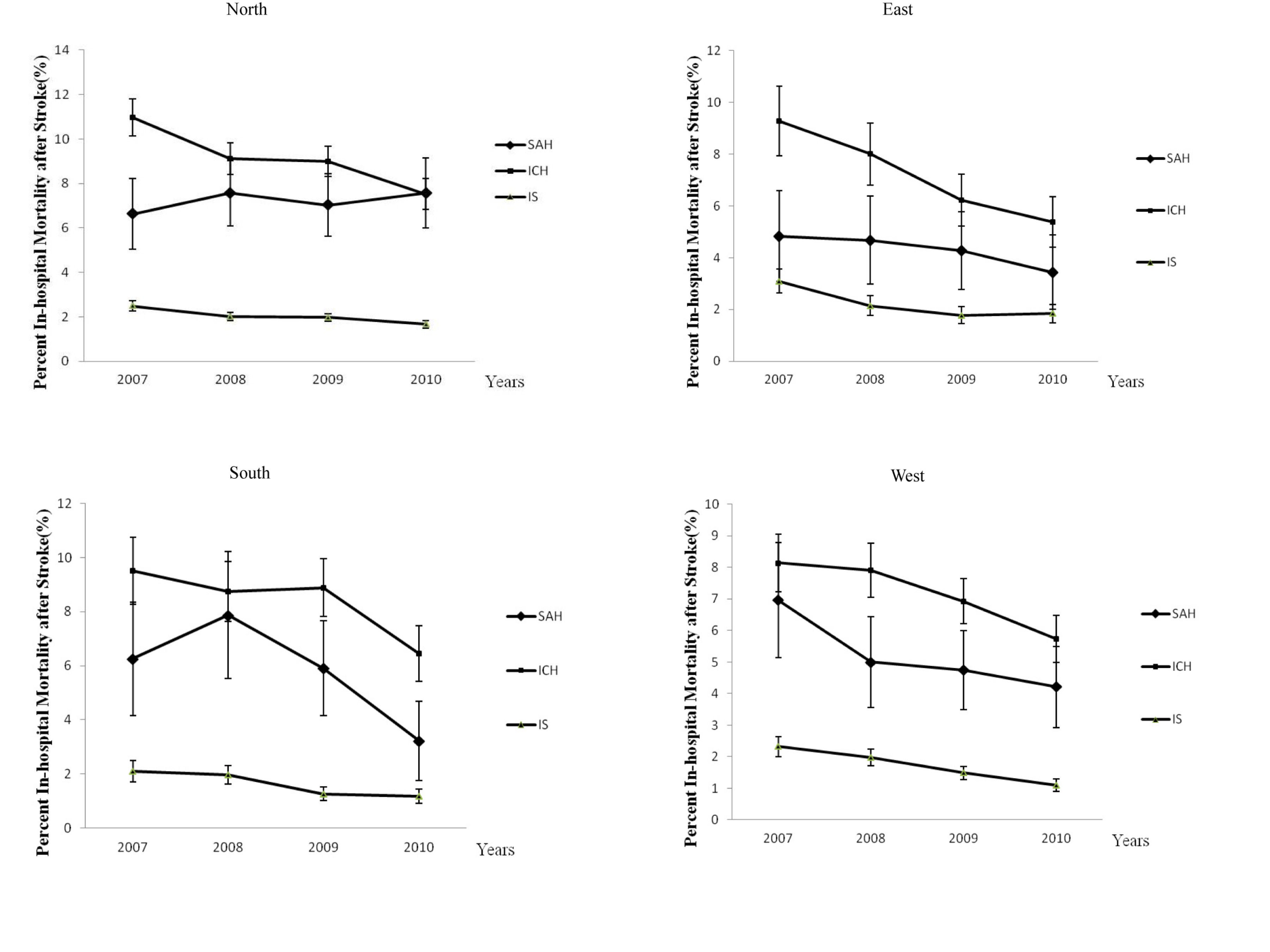

Supplement: Figure S1 — In-Hospital Mortality after Stroke by Type between 2007 and 2010 in the Different Regions. Data presented trends of in-hospital mortality and its 95% CI in different years of three stroke types including SAH, ICH and IS. (TIF) [file pone.0092763.s001.tif]
